# Anxiety, depression, and uncertainty appraisal and factors affecting uncertainty risk and opportunity appraisal of health care workers in Korea during the COVID-19 outbreak: Uncertainty risk and opportunity appraisal

**DOI:** 10.1080/21642850.2023.2182306

**Published:** 2023-02-24

**Authors:** Soo Young An, Jong Sun Ok, Hyeongsu Kim

**Affiliations:** a Department of Emergency Department, Konkuk University Medical Center, Seoul, Republic of Korea; b Department of Nursing, Konkuk University, Chungju-si, Republic of Korea; c Department of Preventive Medicine, School of Medicine, Konkuk University, Seoul, Republic of Korea

**Keywords:** Uncertainty, risk, opportunity, health care workers, COVID-19

## Abstract

**Background:**

Due to the prolonged period of COVID-19, the uncertainty related to COVID-19 is bound to increase for healthcare workers (HCWs) in tertiary medical institutions as much as for the HCWs in dedicated hospitals.

**Purpose:**

To assess anxiety, depression, and uncertainty appraisal, and to determine the factors affecting uncertainty risk and opportunity appraisal experienced by HCWs at the forefront of COVID-19 treatment.

**Method:**

This was a descriptive, cross-sectional study. The participants were HCWs at a tertiary medical center in Seoul. HCWs included medical (doctors, nurses) and non-medical (nutritionists, pathologists, radiologists, office workers, etc.) personnel. Self-reported structured questionnaires (patient health questionnaire, generalized anxiety disorder scale, and uncertainty appraisal) were obtained. Finally, responses from 1337 people were used to evaluate factors affecting uncertainty risk and opportunity appraisal using a quantile regression analysis.

**Results:**

The average ages of medical and non-medical HCWs were 31.69 ± 7.87 and 38.66 ± 11.42 years, and the proportion of females was high. The rates of moderate to severe depression (23.23%) and anxiety (6.83%) were higher in medical HCWs. The uncertainty risk score was higher than the uncertainty opportunity score for all the HCWs. Factors that increased uncertainty opportunity were a decrease in depression in medical HCWs and a decrease in anxiety in non-medical HCWs. Increase in age was directly proportional to uncertainty opportunity in both groups.

**Conclusion:**

There is a need to devise a strategy to reduce uncertainty among HCWs who inevitably face various infectious diseases that appear in the near future. In particular, since there are various types of non-medical as well as medical HCWs in medical institutions who can prepare an intervention plan that comprehensively considers the characteristics of each occupation and the distribution of risks and opportunities of uncertainty will be able to improve the quality of life of HCWs and further promote the health of the people.

## Introduction

In November 2019, the coronavirus disease (COVID-19) was first reported in Wuhan, China. COVID-19 has since become a global health emergency in the form of a global pandemic (World Health Organization, 2020). In the early days of COVID-19, the number of infected people was overwhelmingly high because of overseas inflows, but now sporadic community-based infections continue (Korea Disease Control and Prevention Agency, 2021). As COVID-19 continues, it is a reality that not only health care workers (HCWs) in dedicated hospitals who focus on treating patients infected with COVID-19, but other types of HCWs are also likely to come into contact with COVID-19 patients. In particular, tertiary medical institutions in Seoul are conducting tests at COVID-19 screening clinics, and because of the nature of these institutions, they have no choice but to treat COVID-19 patients at any time in outpatient and emergency rooms. In the United States, Italy, China, Spain, and France, the COVID-19 infection rate among HCWs caring for COVID-19 patients is reported to range from approximately 15%−20% (Ali et al., 2020a; Ali et al., 2020b). Therefore, unlike HCWs in dedicated hospitals that focus on treating patients infected with COVID-19, the uncertainty of when HCWs in tertiary medical institutions may see COVID-19 patients is further increased. Uncertainty, introduced by Mishel, is a variable that affects recovery from disease and is defined as a cognitive state that can be created by disease-related events that are experienced for the first time, unpredictable symptoms, treatment effects that are not guaranteed to be recoverable, and lack of information about the disease processes (Mishel & Braden, 1988). Uncertainty is evaluated in various forms because it is considered an important factor not only for patients with chronic diseases (Kang, 2009; Kim & Hong, 2018) but also for parents and families who manage chronic diseases together (Arias-Rojas et al., 2019; Connolly et al., 2021; Yoo, 2007). COVID-19 contains all the characteristics of uncertainty suggested by Mishel, and HCWs at the forefront of patient care can be considered representative groups involved in direct patient care, similar to the parents and family members of patients with chronic diseases. The appraisal of uncertainty is a cognitive thinking process that provides meaning to uncertainty risk or opportunity. If uncertainty is evaluated as a risk to an individual, it will lead to negative results; on the contrary, if it is evaluated as an opportunity, it can lead to the establishment of a new outlook on life because the ability to cope with diseases is correspondingly improved. Uncertainty is inevitably influenced by various physical and psychological factors. Due to COVID-19, HCWs work long hours in stressful work environments and complain of fatigue and isolation (Ali et al., 2020b).

In HCWs, the anxiety rate was 30.5% (Adibi et al., 2021) and the depression rate was 31.1% (Marvaldi et al., 2021). The rates of sleep disorder and insomnia were 44.0% (Marvaldi et al., 2021) and 36.36% (Sahebi et al., 2021), respectively.

Since HCWs include both medical (doctors and nurses) and non-medical (assistants, nutritionists, and radiologists) HCWs, a comprehensive understanding of them is required. Moreover, the goals of disease prevention, treatment, and health promotion of patients can be achieved only when various HCWs cooperate. However, previous studies have mainly focused on medical HCWs (Buselli et al., 2020; Domínguez-Salas et al., 2021). Therefore, this study investigated various physical and psychological factors that can affect uncertainty risk and opportunity appraisal according to the degree of distribution of uncertainty for both medical and non-medical HCWs. Thus, we collected basic data for the development of intervention programs that can reduce uncertainty risks and enhance uncertainty opportunities by considering professional and personal characteristics of various HCWs.

## Methods

### Study participants

The participants were HCWs involved in the treatment and management of COVID-19 patients in Korea. at a tertiary medical center. Medical HCWs include doctors and nurses, and non-medical HCWs include assistants and transfer agents, nutritionists, clinical pathologists, radiologists, physical therapists, office workers, and computer administrators. It is estimated that the hospital has approximately 2,000 direct and indirectly employed staff, out of which, 1500 questionnaires were distributed to departments that obtained cooperation. Among them, 1384 questionnaires were collected (92.3% response rate), 47 people who did not properly respond to the questionnaire were excluded, and data from 1337 people were finally analyzed.

### Study design

This cross-sectional study was designed to assess anxiety, depression, and uncertainty appraisal, and to determine the factors affecting uncertainty risk and opportunity appraisal experienced by HCWs at the forefront of COVID-19 treatment.

### Data collection and research ethics

The data collection period for this study was from October to November 2020. Before proceeding with the study, data collection was performed with the approval of the institutional review board (IRB 7001355-202010-HR-406) of Konkuk University.

The purpose of the questionnaire was explained to HCWs using an online bulletin board, and participants were encouraged to participate in the questionnaire. Afterward, a self-reported structured questionnaire and written consent forms were distributed to each department, and cooperation from each department was obtained. The investigator finally collected the questionnaires and consent forms from each department.

### Measurements

#### General characteristics

The general characteristics of the subjects, such as age, sex, occupation, marital status, and working hours per day, were investigated.

#### Physical health

The physical health of the participants, such as exercise, drinking, smoking, subjective health status (SHS), status of daily living scores (SDLS), and physical burden was investigated. Regular exercise (more than three times a week, to the point of sweating for at least 30 min), whether they drank alcohol regularly, or had smoked more than five packs of cigarettes (100 cigarettes) in their life so far (smoker, past smoker, non-smoker) was also investigated. The SHS uses a five-point Likert scale, with a score of 1 being ‘very good’ and 5 being ‘very bad,’ with a higher score indicating a negative perception of SHS. SDLS is divided into 10-point intervals ranging from 0 to 100, with a higher score indicating that daily living is not impaired compared to the pre-COVID-19 outbreak. Physical burden uses a five-point Likert scale, with a score of 1 being ‘not at all difficult’ and 5 being ‘very difficult,’ with higher scores indicating a greater physical burden.

#### Psychological health

The psychological health of the subjects, such as psychological burden, depression, anxiety, and uncertainty, was investigated. For psychological burden, a five-point Likert scale was used, with a score of 1 being ‘not at all difficult’ and 5 being ‘very difficult,’ with higher scores indicating a greater psychological burden. To measure the degree of depression, a nine-item patient health questionnaire scale (Kocalevent et al., 2013) was used. A translation and a back-translation method were used, and approved by its author (Park et al., 2010). Subjects were asked how often they experienced by each of the depressive symptoms over the last two weeks. The scores were 0 (not at all), 1 (several days), 2 (more than half the days), and 3 (nearly every day). The cutoff score of this tool is 10 points, and no/minimal depression is 0–4 points, mild depression is 5–9 points, moderate depression is 10–14 points, and severe depression is 15–27 points. For criterion validity, sensitivity and specificity were .88, and Cronbach’s α reliability was .86-.89. The test-retest reliability was .84 and the area under the curve was .95 (Kroenke et al., 2001).

To measure the degree of anxiety, a seven-item generalized anxiety disorder scale (Löwe et al., 2008) was used. A translation and a back-translation method were used, and approved by its author (Lee et al., 2022). Subjects were asked how often they experienced by each of the seven anxiety symptoms during the last two weeks. Scores of 0 (not at all), 1 (several days), 2 (more than half the days), and 3 (nearly every day) were assigned. The cutoff score of this tool was 7 points: no/minimal anxiety, 0–4 points; mild anxiety, 5–9 points; moderate anxiety, 10–14 points; and severe anxiety, 15–21 points. In criterion validity, sensitivity was .89, specificity was .82, and the area under the curve was .91 (Spitzer et al., 2006). The Cronbach’s alpha for internal consistency was. 89 for the public (Löwe et al., 2008). The tool developed for uncertainty appraisal (Mishel & Sorenson, 1991) was used. A translation and a back-translation method were used, and approved by its author (Cha & Kim, 2012). Using uncertainty appraisal, we investigated how individuals felt about the ongoing COVID-19 pandemic. The uncertainty appraisal scale is divided into uncertainty risk and uncertainty opportunity. The eight items for uncertainty risk included anger, worry, sadness, anxiety, disappointment, fear, guilt, and boredom. The seven items for uncertainty opportunity included feeling good, confidence, satisfaction, hope, anticipation, euphoria, and relief. The scores were 0 (not at all), 1 (hardly not), 2 (a few), 3 (somewhat), 4 (a lot), and 5 (very much); it uses a six-point Likert scale. Uncertainty risk is a total of 0–40 points, and uncertainty opportunity is a total of 0–35 points. In the study by Mishel and Sorenson, reliability for uncertainty risk’s Cronbach's alpha was .87 and uncertainty opportunity’s Cronbach's alpha was .82 (Mishel & Sorenson, 1991). Both uncertainty risk and opportunity in the study by Cha and Kim had Cronbach’s alpha of .88 (Cha & Kim, 2012).

### Data analysis

The collected data were analyzed using the R statistical program (R Foundation for Statistical Computing, Vienna, Austria). Descriptive statistics on general characteristics and physical and psychological health were displayed as means and standard deviations, or frequencies and percentages. In this study, uncertainty risk and opportunity were evaluated using quantile regression analysis, rather than classical regression analysis. Unlike classical regression analysis, which estimates determinants based on the conditional mean of the dependent variable, quantile regression analysis can be used when the determinants of the dependent variable for each quantile differ based on the conditional distribution of the dependent variable (Park et al., 2018). In other words, quantile regression analysis is a method for estimating the relationship between an explanatory variable and a dependent variable by obtaining the conditional quantile of the dependent variable. Therefore, when the least-squares method is used, if normality and homoscedasticity are violated, it can be controlled. Furthermore, compared to the least-squares method, it is a very useful statistical method in that it can estimate various distributions of the response variable to the explanatory variable. For missing values, the multiple imputation method was applied on the premise of random missing values ⁣⁣for the questionnaire content. The multiple imputation method is an analysis method that creates several values that are ⁣⁣estimated to be real values ⁣⁣using existing data while preserving the uncertainty of missing values (Sterne et al., 2009). This method can use complete data without discarding missing values and can use uncertainty information about the substituted values. Additionally, compared with the single imputation method, the multiple imputation method has the advantage of increasing the efficiency of estimation by repeating random substitutions based on the data distribution and then summing them. In this study, the repletion of the algorithm was 10 times.

## Results

### General characteristics

In the general characteristics (Table 1), the average ages of medical and non-medical HCWs were 31.69 ± 7.87 and 38.66 ± 11.42 years, respectively. The proportion of females was higher among both medical and non-medical HCWs. Non-medical HCWs had a higher percentage of married people (53.01%), while medical HCWs had a higher percentage of others (67.85%). The daily working hours of medical and non-medical HCWs were 9.17 ± 2.92, and 8.84 ± 3.60 h, respectively.

**Table 1.** Characteristics of subjects.

	N = 1,377			
	Categories	Total	Medical healthcare workers	Non-medical healthcare workers
		n(%) or M ± SD	n(%) or M ± SD	n(%) or M ± SD
General characteristics	Age	34.80 ± 10.22	31.69 ± 7.87	38.66 ± 11.42
	Sex			
	Male	262(19.03)	22(2.89)	240(39.02)
	Female	1115(80.97)	740(97.11)	375(60.98)
	Marital status			
	Married	571(41.47)	245(32.15)	326(53.01)
	Others*	806(58.53)	517(67.85)	289(46.99)
	Working time (hr/day)	9.02 ± 3.25	9.17 ± 2.92	8.84 ± 3.60
Physical health	Exercise			
	Yes	355(25.78)	158(20.74)	197(32.03)
	No	1022(74.22)	604(79.27)	418(67.97)
	Drinking			
	No	628(45.61)	346(45.41)	282(45.85)
	Yes	749(54.39)	416(54.59)	333(54.15)
	Smoking			
	Nonsmoker	1233(89.54)	752(98.69)	481(78.21)
	Past smoker	64(4.65)	5(0.66)	59(9.59)
	Smoker	80(5.81)	5(0.66)	75(12.20)
	SHS			
	Very good	61(4.43)	24(3.15)	37(6.02)
	Good	381(27.67)	181(23.75)	200(32.52)
	Usually	772(56.06)	451(59.19)	321(52.20)
	Bad	155(11.26)	100(13.12)	55(8.94)
	Very bad	8(0.58)	6(0.79)	2(0.33)
	SDLS			
	0	10(0.73)	6(0.79)	4(0.65)
	10	47(3.41)	33(4.33)	14(2.28)
	20	155(11.26)	104(13.65)	51(8.29)
	30	282(20.48)	192(25.20)	90(14.63)
	40	175(12.71)	98(12.86)	77(12.52)
	50	319(23.17)	153(20.08)	166(26.99)
	60	151(10.97)	83(10.89)	68(11.06)
	70	140(10.17)	63(8.27)	77(12.52)
	80	65(4.72)	21(2.76)	44(7.15)
	90	18(1.31)	6(0.79)	12(1.95)
	100	15(1.10)	3(0.39)	12(1.95)
	Physical burden			
	Not hard at all	32(2.32)	9(1.18)	23(3.74)
	Not hard	138(10.02)	53(6.96)	85(13.82)
	Usually	462(33.55)	195(25.59)	267(43.42)
	Hard	564(40.96)	374(49.08)	190(30.89)
	Very hard	181(13.15)	131(17.19)	50(8.13)
Psychological health	Psychological burden			
	Not hard at all	15(1.10)	2(0.26)	13(2.11)
	Not hard	66(4.79)	18(2.36)	48(7.81)
	Usually	503(36.53)	172(22.57)	331(53.82)
	Hard	540(39.22)	361(47.38)	179(29.11)
	Very hard	253(18.37)	209(27.43)	44(7.15)
	Depression	5.98 ± 4.79	6.90 ± 4.92	4.81 ± 4.31
	No/minimal	603(43.79)	260(34.12)	343(55.77)
	Mild	519(37.69)	325(42.65)	194(31.55)
	Moderate	178(12.93)	119(15.62)	59(9.59)
	Severe	77(5.59)	58(7.61)	19(3.09)
	Anxiety	2.93 ± 3.69	3.32 ± 3.92	2.46 ± 3.33
	Minimal	1047(76.04)	552(72.44)	495(80.49)
	Mild	251(18.23)	158(20.74)	93(15.12)
	Moderate	46(3.34)	28(3.68)	18(2.93)
	Severe	33(2.40)	24(3.15)	9(1.46)
	Uncertainty appraisal			
	Risk	15.47 ± 7.30	16.16 ± 7.04	14.63 ± 7.53
	Opportunity	10.26 ± 6.84	9.31 ± 6.41	11.43 ± 7.17

Others* = single, divorced, or widowed; SHS = Subjective health status; SDLS = Status of daily living score.

### Physical health

Regarding physical health (Table 1), the ratio of not exercising regularly was higher than that of exercising regularly in both groups, as was the ratio of non-smokers. In the case of drinking, the drinking rate was > 50% in both groups. Regarding SHS, the proportion of respondents who answered that their SHS was ‘bad’ or ‘very bad’ was 13.91% for medical HCWs, and 9.27% for non-medical HCWs. The percentage of SDLS less than 50 was higher (48.59%) among medical HCWs than among non-medical HCWs (38.37%). Regarding physical burden, the proportion of respondents who answered ‘difficult’ or ‘very difficult’ was 39.02% for non-medical HCWs and 66.27% for medical HCWs.

### Psychological health

Regarding psychological health (Table 1), 36.26% of non-medical HCWs answered that their psychological burden was ‘difficult’ or ‘very difficult’, compared to 74.81% of medical HCWs. The average scores for depression and anxiety were 6.90 ± 4.92 and 3.32 ± 3.92 points for medical HCWs, and 4.81 ± 4.31 and 2.46 ± 3.33 points for non-medical HCWs, respectively. The rates of moderate to severe depression and anxiety were 23.23% and 6.83% for medical HCWs and 12.68% and 4.39% for non-medical HCWs, respectively. The uncertainty risk and opportunity scores of medical HCW were 16.16 ± 7.04 and 9.31 ± 6.41 points, and 14.63 ± 7.53 and 11.43 ± 7.17 points for non-medical HCWs, respectively.

### Factors affecting uncertainty risk and opportunity appraisal

**Figure 1.**
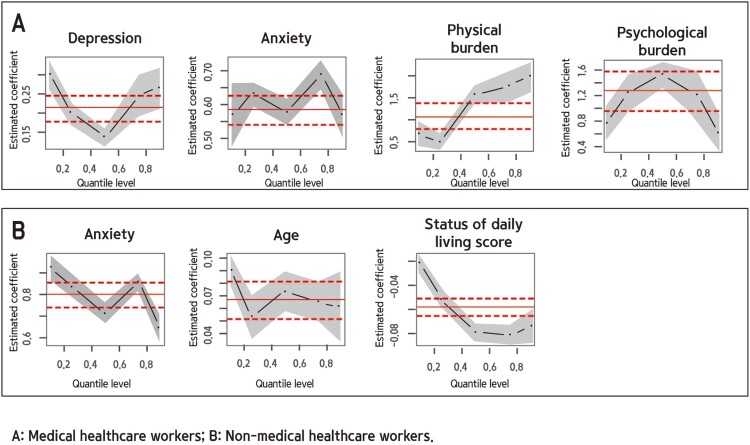
Factors affecting the uncertainty risk appraisal.

Factors affecting uncertainty risk appraisal are shown in Table 2 and Figure 1. Increases in depression and anxiety scores and physical and psychological burden among medical HCWs were found to increase the uncertainty risk. Among non-medical HCWs, increased anxiety scores and age and decreased SDLS were found to increase the uncertainty risk.

**Table 2.** Uncertainty risk appraisal of medical and non-medical HCWs.

	Uncertainty risk appraisal of medical healthcare workers					Uncertainty risk appraisal of non-medical healthcare workers				
Items	0.1(*p*)	0.25(*p*)	0.5(*p*)	0.75(*p*)	0.9(*p*)	0.1(*p*)	0.25(*p*)	0.5(*p*)	0.75(*p*)	0.9(*p*)
Depression	0.297***	0.201***	0.140***	0.244***	0.264***	0.201***	0.203***	0.155***	0.012	−0.088*
Anxiety	0.568***	0.631***	0.575***	0.687***	0.570***	0.922***	0.836***	0.714***	0.855***	0.648***
Age	−0.008	0.021**	−0.008	0.022***	−0.073***	0.091***	0.054***	0.074***	0.065***	0.062***
Spouse	0.060	0.870***	1.632***	1.124***	2.026***	0.545**	0.792***	−0.104	−0.766***	−0.104
WT(hr/day)	0.000	−0.016***	−0.024	−0.017	−0.017**	0.000	−0.006	0.001	−0.030*	−0.060***
SDLS	−0.066***	−0.038***	−0.018***	−0.011**	−0.005	−0.021***	−0.051***	−0.078***	−0.081***	−0.073***
SHS(bad)	−0.105	0.458*	1.483***	0.959***	2.046**	2.985***	2.126***	0.382	2.192***	2.366*
Exercise	−0.257	−0.028	0.487***	0.338	0.346	−1.625***	−2.046***	−0.927***	0.183	−0.036
Drinking	−0.123	0.426**	0.027	−0.131	−0.151	0.781***	0.039	−0.130	−0.100	−0.156
Smoking	4.424	4.250***	6.439***	3.073	3.155	0.211	−1.216***	−1.448***	−1.046**	−0.081
PhysiB	0.665***	0.486***	1.562***	1.759***	1.963***	−0.035	−0.219	1.054***	0.963***	0.867*
PsychoB	0.755***	1.227***	1.506***	1.202***	0.601***	0.944***	1.798***	0.340	0.322	0.487

Spouse = Having spouse; WT = working time; SDLS = Status of daily living score; SHS(bad) = Subjective health status(bad); PhysiB = Physical burden; PsychoB = Psychological burden

***p < .001, **p < .01, *p < .05

0.1 = 10TH quantile; 25 = 25th quantile; 75 = 75th quantile; 9 = 90th quantile

**Figure 2.**
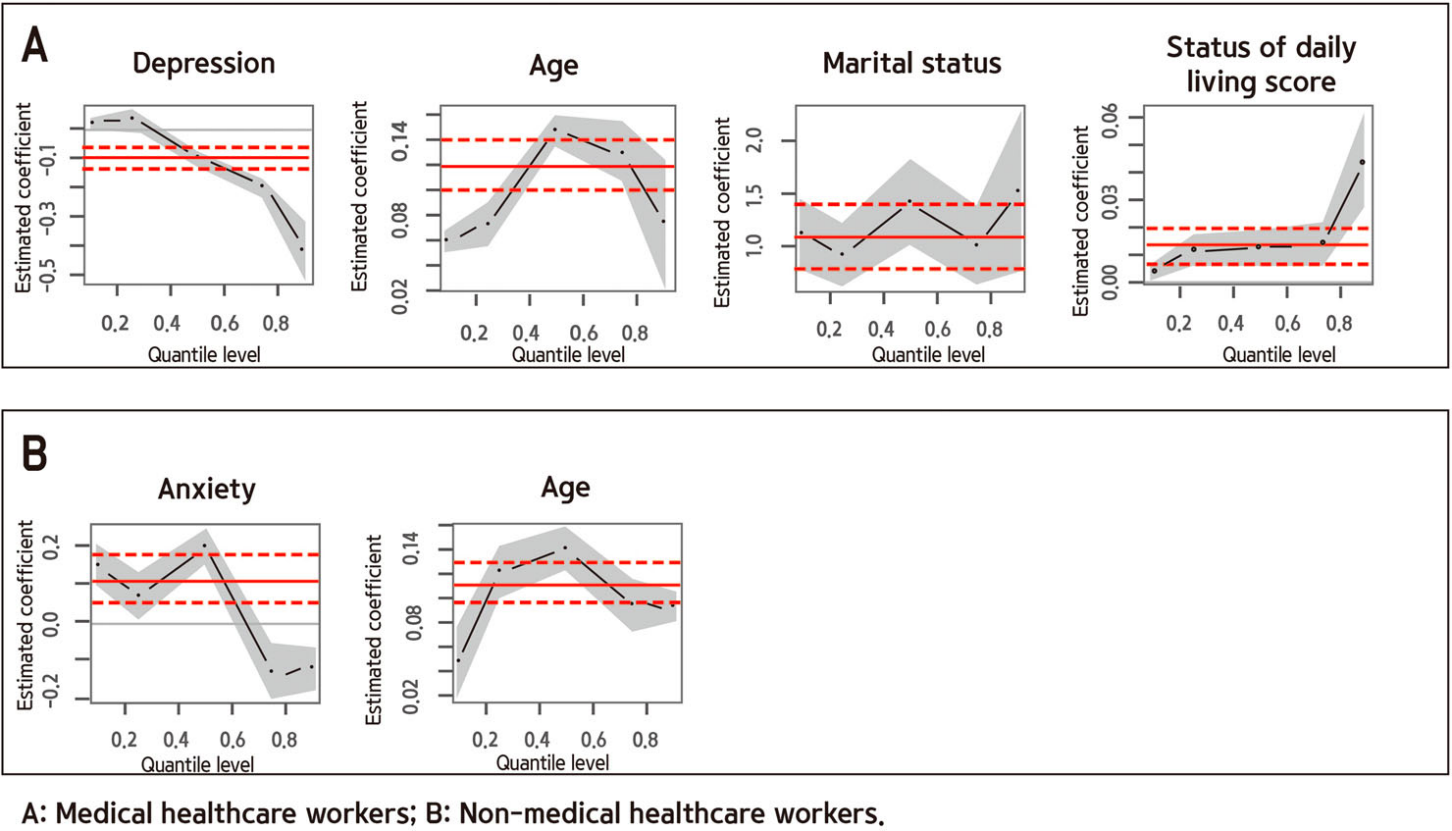
Factors affecting the uncertainty opportunity appraisal.

Factors affecting uncertainty opportunity appraisal are shown in Table 3 and Figure 2. Among medical HCWs, increasing age, having a spouse, and increasing SDLS were found to improve uncertainty opportunities. The depression score was high among medical HCWs with a low uncertainty opportunity score and low with a high uncertainty opportunity score. Among non-medical HCWs, increasing age was found to improve uncertainty opportunities. The anxiety score was high among non-medical HCWs with a low uncertainty opportunity score and low with a high uncertainty opportunity score.

**Table 3.** Uncertainty opportunity appraisal of medical and non-medical HCWs.

	Uncertainty opportunity appraisal of medical healthcare workers					Uncertainty opportunity appraisal of non-medical healthcare workers				
Items	0.1(*p*)	0.25(*p*)	0.5(*p*)	0.75(*p*)	0.9(*p*)	0.1(*p*)	0.25(*p*)	0.5(*p*)	0.75(*p*)	0.9(*p*)
Depression	0.023***	0.035*	−0.100***	−0.200***	−0.414***	0.061	0.012	−0.249***	−0.290***	−0.393***
Anxiety	0.012	−0.037	0.038	−0.023	−0.005	0.148***	0.066*	0.195***	−0.130***	−0.121***
Age	0.061***	0.075***	0.150***	0.131***	0.075*	0.047**	0.121***	0.141***	0.094***	0.093***
Spouse	1.110***	0.931***	1.411***	1.008***	1.513***	2.417***	2.322***	1.378***	1.472***	0.007
WT(hr/day)	−0.025***	0.022***	0.022	−0.017	0.007	−0.002	0.043*	0.015	−0.015	−0.007
SDLS	0.004*	0.012***	0.013***	0.014**	0.044***	−0.007	−0.027***	−0.011*	−0.012*	0.049***
SHS(bad)	−0.753***	−1.283***	−0.576*	−0.694*	0.636	0.812	−0.268	−1.426***	−0.423	0.244
Exercise	0.391	0.767***	1.096***	0.875***	1.252**	0.013	0.184	0.948***	0.263	1.511***
Drinking	−0.939***	−0.318*	−0.270	−0.191	−0.647	0.168	0.337	0.513*	−0.359	−0.845***
Smoking	−3.716***	−0.841	−1.398	−1.657	−0.362	0.500	1.722***	0.872**	0.845*	0.722
PhysiB	0.046	−0.279*	0.174	0.850***	1.290*	−0.489*	0.289	0.050	−1.580***	−1.353***
PsychoB	0.029	−0.733***	−1.276***	−1.656***	−3.291***	0.752***	−0.251	0.053	0.549*	0.447**

Spouse = Having spouse; WT = Working time; SDLS = Status of daily living score; SHS(bad) = Subjective health status(bad); PhysiB = Physical burden; PsychoB = Psychological burden

***p < .001,**p < .01,*p < .05

0.1 = 10TH quantile; 25 = 25th quantile; 75 = 75th quantile; 9 = 90th quantile

## Discussion

This study investigated the factors affecting uncertainty risk and opportunity for both medical HCWs and non-medical HCWs, with different influencing factors for both.

This study found that the uncertainty risk score was higher than the uncertainty opportunity scores for all HCWs; in particular, the uncertainty risk score of medical HCWs was higher than that of non-medical HCWs. In the case of patients with cancer, coronary artery disease, and heart failure, the uncertainty opportunity score was higher than the uncertainty risk score. Although there were differences by disease, the uncertainty risk scores ranged from 8.3–20.5, and the uncertainty opportunity scores range from 10.3–24.2 points (Cha & Kim, 2012; Lee, 2009; Lee, 2010; Lee & Kang, 2020). HCWs had a higher uncertainty risk but lower uncertainty opportunity than patients with chronic diseases. In this study, factors that increased uncertainty risk increased depression and anxiety among medical HCWs and increased anxiety among non-medical HCWs. Conversely, factors that increased uncertainty opportunity were a decrease in depression among medical HCWs and a decrease in anxiety among non-medical HCWs. To reduce uncertainty risk and increase uncertainty opportunity, there is a need for psychological intervention methods that can reduce anxiety and depression in both medical and non-medical HCWs. According to a previous systematic review (Salari et al., 2020), the rates of anxiety and depression among hospital staff (excluding doctors or nurses) during the COVID-19 pandemic were 27% and 20.6%, respectively. Furthermore, the incidence rates of anxiety and depression were 19.8% and 40.4% for doctors, and 22.8% and 28% for nurses, respectively. Doctors and nurses had a higher rate of depression than anxiety, while other hospital staff members had higher anxiety scores. In this study, the rate of moderate to severe depression was nearly twice as high in medical HCWs than in non-medical HCWs, which was consistent with the results of previous studies. However, the rate of moderate to severe anxiety was higher in medical HCWs than in non-medical HCWs, which was contradictory to the results of previous studies. Nevertheless, anxiety was an important factor that increased uncertainty risk among non-medical HCWs in this study. According to previous research, the reasons for the anxiety of non-medical HCWs were low access to official psychological support and lack of direct medical information about the onset of infectious diseases (Tan et al., 2020). Previous studies have suggested various interventions to reduce anxiety and depression; in particular, relaxation training (Teixeira et al., 2005), cognitive–behavioral therapy, and interpersonal therapies have been suggested as psychological interventions (Fava, 2003; Raes et al., 2009). Because medical professionals are a group with a stronger sense of responsibility and calling than anyone else, what is important to them is colleagues who can empathize with and encourage them. In particular, the protection of patients’ personal information is an important issue for HCWs; therefore, the activation of online and offline communities within medical institutions for HCWs should be considered. Through this, HCWs in medical institutions could have the opportunity to share their opinions and empathize with and encourage each other. Medical HCWs face the suffering and death of patients in their field (Shay, 2014). However, in the context of COVID-19, many HCWs reported experiencing moral injuries (Walton et al., 2020). Before the COVID-19 pandemic, family, friends, and religious people could participate in a patient’s death process, and a funeral was a time to share their grief and accept death. However, during the COVID-19 pandemic, it has become impossible to carry out all of these processes. Consequently, HCWs face various ethical dilemmas. Therefore, it is necessary to form small focus or leader-centered groups to share and discuss the moral injury experiences of HCWs and regularly exchange opinions and information with each other.

The findings of this study revealed that the factors that increased uncertainty risk were increased physical burden for medical HCWs and decreased SDLS for non-medical HCWs. This suggests that not only psychological interventions but also physical interventions are necessary to lower uncertainty risk and increase uncertainty opportunity. Previous studies have reported that the psychological burden experienced by medical HCWs can be expressed by a variety of physical symptoms (Chew et al., 2020). The proportion of HCWs participating in regular exercise was quite low, and the rate of drinking was 50% for HCWs in this study. Therefore, a physical intervention method for HCWs is necessary; in particular, an intervention method with fewer time and space restrictions should be considered. Time and frequency of exercise and stretching could be specified; for example, performing exercises using a hand grip or stretching band in the work schedule of HCWs. Balanced nutrition is the most important factor in maintaining physical health. However, it is not easy to secure mealtime, and even if time allows it, HCWs often have to eat a lunch box or instant food in a hurry. In particular, time and physical restrictions because of wearing level D protective clothing per the COVID-19 quarantine guidelines and the shortage of manpower because of the increase in the COVID-19 infection rates among fellow HCWs exacerbates this situation. Accordingly, increasing the extra pay for HCWs may be considered. As the work of HCWs because of the COVID-19 pandemic has been overburdened for a long time, we expect positive results from discussions by the government about an increase in extra pay for HCWs.

An increase in age increased uncertainty opportunity in both medical and non-medical HCWs in this study. An increase in age in the medical field implies that they have various work experiences and effective communication skills. Therefore, it is necessary to establish a medical work environment in which HCWs with various experiences can continue working. In reality, most non-medical HCWs are contract or temporary workers. To guarantee employment stability and increase work efficiency of medical institutions, it may be possible to consider a method of providing additional points to those who have experience in managing infectious diseases when evaluating their work ability for reemployment. Furthermore, it is necessary to allow HCWs to share diverse knowledge and experiences through mentor-mentee programs with senior groups. Consequently, the senior group will be recognized for their worth and more satisfaction with their lives, and many HCWs who experience new infectious diseases will be able to work with the confidence that they have a good human support system.

This study differs from others in that it not only considered potential risks in the medical field by taking an interest in uncertainty risk but also took an interest in the uncertainty opportunities for the growth potential of HCWs. Nevertheless, this study has some limitations and based on these, suggestions are made. First, it is necessary to be careful in generalizing the results of the study because information on HCWs from only one medical institution was collected. In particular, most medical HCWs were nurses, and the proportion of women among both medical and non-medical HCWs was relatively high. Therefore, it is necessary to further expand research institutions and subjects in consideration of the size and region of medical institutions and the gender and occupation of HCWs in the future.

The results of this study suggest that there is a need to devise a strategy to reduce uncertainty among HCWs who inevitably face various infectious diseases that are expected to appear in the near future. Because there are various types of non-medical and medical HCWs, preparing an intervention plan that comprehensively considers the characteristics of each occupation and the distribution of risks and opportunities of uncertainty will help improve the quality of life of HCWs and further promote health of the people.

## Conclusion

This study is meaningful in that it investigated factors affecting uncertainty risks and opportunities for medical and non-medical HCWs working during the COVID-19 pandemic. Medical HCWs who spent a long time in direct contact with patients had a higher uncertainty risk than non-medical HCWs but a relatively low uncertainty opportunity. Among medical HCWs, it was found that an increase in depression and anxiety and an increase in physical and mental burdens raised uncertainty risk. Among non-medical HCWs, an increase in anxiety score and age, as well as a decrease in the SDLS, were found to increase the uncertainty risk. Among medical HCWs, decreased depression, increased age, having a spouse, and increased SDLS were found to improve uncertainty opportunity. Among non-medical HCWs, a decrease in anxiety and an increase in age were found to improve uncertainty opportunity. These results underscore the importance of thoroughly preparing differentiated intervention strategies for various HCWs to successfully perform their roles in the medical field for the treatment of COVID-19.

## Data Availability

All the data and methods of statistical analysis were included in the manuscript.
